# Metabolomic analyses reveal lipid abnormalities and hepatic dysfunction in non-human primate model for *Yersinia pestis*

**DOI:** 10.1007/s11306-018-1457-2

**Published:** 2018-12-29

**Authors:** Aarti Gautam, Seid Muhie, Nabarun Chakraborty, Allison Hoke, Duncan Donohue, Stacy Ann Miller, Rasha Hammamieh, Marti Jett

**Affiliations:** 1 US Army Center for Environmental Health Research, 568 Doughten Drive, Fort Detrick, MD 21702 USA; 2 The Geneva Foundation, Fort Detrick, MD USA

**Keywords:** *Yersinia pestis*, Metabolomics, Non-human primate, Pneumonic plague, Animal model

## Abstract

**Introduction:**

Pneumonic plague is caused by the aerosolized form of *Yersinia pestis* and is a highly virulent infection with complex clinical consequences, and without treatment, the fatality rate approaches 100%. The exact mechanisms of disease progression are unclear, with limited work done using metabolite profiling to study disease progression.

**Objective:**

The aim of this pilot study was to profile the plasma metabolomics in an animal model of *Y. pestis* infection.

**Methods:**

In this study, African Green monkeys were challenged with the highly virulent, aerosolized *Y. pestis* strain CO92, and untargeted metabolomics profiling of plasma was performed using liquid and gas chromatography with mass spectrometry.

**Results:**

At early time points post-exposure, we found significant increases in polyunsaturated, long chain fatty acid metabolites with *p* values ranging from as low as 0.000001 (ratio = 1.94) for the metabolite eicosapentaenoate to 0.04 (ratio = 1.36) for the metabolite adrenate when compared to time-matched controls. Multiple acyl carnitines metabolites were increased at earlier time points and could be a result of fatty acid oxidation defects with *p* values ranging from as low as 0.00001 (ratio = 2.95) for the metabolite octanoylcarnitine to 0.04 (ratio = 1.33) for metabolite deoxycarnitine when compared to time-matched controls. Dicarboxylic acids are important metabolic products of fatty acids oxidation, and when compared to time matched controls, were higher at earlier time points where metabolite tetradecanedioate has a ratio of 4.09 with significant *p* value of 0.000002 and adipate with a ratio of 1.12 and *p* value of 0.004. The metabolites from lysolipids (with significant *p* values ranging from 0.00006 for 1-oleoylglycerophosphoethanolamine to 0.04 for 1-stearoylglycerophosphoethanolamine and a ratio of 0.47 and 0.78, respectively) and bile acid metabolism (with significant *p* values ranging from 0.02 for cholate to 0.04 for deoxycholate and a ratio of 0.39 and 0.66, respectively) pathways were significantly lower compared to their time-matched controls during the entire course of infection. Metabolite levels from amino acid pathways were disrupted, and a few from the leucine, isoleucine and valine pathway were significantly higher (*p* values ranging from 0.002 to 0.04 and ratios ranging from 1.3 to 1.5, respectively), whereas metabolites from the urea cycle, arginine and proline pathways were significantly lower (*p* values ranging from 0.00008 to 0.02 and ratios ranging from 0.5 to 0.7, respectively) during the course of infection.

**Conclusions:**

The involvement of several lipid pathways post-infection suggested activation of pathways linked to inflammation and oxidative stress. Metabolite data further showed increased energy demand, and multiple metabolites indicated potential hepatic dysfunction. Integration of blood metabolomics and transcriptomics data identified linoleate as a core metabolite with cross-talk with multiple genes from various time points. Collectively, the data from this study provided new insights into the mechanisms of *Y. pestis* pathogenesis that may aid in development of therapeutics.

**Electronic supplementary material:**

The online version of this article (10.1007/s11306-018-1457-2) contains supplementary material, which is available to authorized users.

## Introduction

Plague is an infectious disease caused by *Yersinia pestis*, a naturally occurring bacterium, primarily infecting wild rodents and transmitted by fleas. The majority of cases of human plague also result from flea bites, and are of the bubonic form (Perry and Fetherston 1997). Primary pneumonic plague, resulting from inhalation of plague bacteria, rarely occurs under natural conditions (Pechous et al. 2016), but the virulence and ease of aerosolization of *Y. pestis* makes it a potential bioweapon (Krishna and Chitkara 2003), with pneumonic plague being the expected form of disease following an aerosol attack (Pechous et al. 2016; Rollins et al. 2003; Verma and Tuteja 2016).

Humans with primary pneumonic plague initially experience a febrile illness with headaches and chills, followed by a rapid progression to fulminant pneumonia, which, if left untreated, results in death within 72 h post-infection (p.i.) (Inglesby et al. 2000; Perry and Fetherston 1997; Riedel 2005; Smiley 2008). Pneumonic plague is nearly always fatal unless treated with antibiotics within 20 h of onset (Butler 2012). The exceptionally rapid course of pneumonic plague suggests that the virulence of *Y. pestis* in a mammalian host results from inadequate adaptive and innate immune responses (Yang et al. 2017). Many studies support this concept, but few have validated the specific impairment of the host defense in vivo. The information available to date indicates that rodents and non-human primate (NHP) models of pneumonic plague mimic the human disease (Coate et al. 2014; Eddy et al. 2015; Finegold et al. 1968; Hammamieh et al. 2016; Koster et al. 2010; Lathem et al. 2005; Peters et al. 2013; Warren et al. 2011). In NHP models, the robust cellular responses that typically characterize other bacterial pneumonias are delayed and ineffective during pneumonic plague (Finegold 1969; Smiley 2008). Similarly, mouse models reveal steadily progressive bacterial growth in pulmonary tissues, with dissemination to other organs by 36 h p.i. (Bubeck et al. 2007; Lathem et al. 2007). As compared to other Gram-negative bacterial diseases, pulmonary infection that is caused by *Y. pestis* elicits a delayed inflammatory response (Cantwell et al. 2010) that may be attributed to the route of exposure.

Several studies have been carried out in the past to elucidate the host responses to *Y. pestis* infection in the animal model, mainly by transcriptomic and proteomic approaches (Chromy et al. 2005, 2004; Comer et al. 2010; Du et al. 2014; Yang et al. 2017; Zhang et al. 2005). The missing in-depth knowledge of the host metabolic response is due to the fact that this response is a complex phenomenon comprised of multiple steps (Eisenreich et al. 2013). The main focus of the current study is to correlate the *Yersinia*-triggered immune and inflammatory responses with the metabolites identified in plasma. Metabolic profiling can provide a window into instantaneous, as well as long term, physiological or pathological changes as a complement to transcriptomic and proteomic profiling in the systemic and functional studies of living organisms (Mangalam et al. 2013; Tebani et al. 2016). Metabolomics has contributed greatly to our understanding of the actions of pharmaceutical agents (Beger et al. 2016) and to the diagnosis of chronic and infectious diseases (Banoei et al. 2017; Shommu et al. 2015; Voge et al. 2016). This can help us to advance the understanding of disease mechanisms and to improve disease diagnostics, as indicated recently for ionizing radiation response (Johnson et al. 2012), and to further interpret the metabolic pathways of lung injury in mice (Cui et al. 2016). The current study characterized the longitudinal profiling of plasma metabolites in an NHP model of *Yersinia* infection and is a follow-up to the previous study where we investigated the transcriptome profile of the blood collected from this animal model (Hammamieh et al. 2016). This study displays the usefulness of metabolomics to track the progression of metabolic changes over time in NHPs infected with *Y. pestis*.

## Materials and methods

### Ethics statement

All animal experiments were approved by the Institutional Animal Care and Use Committee (IACUC) at the Walter Reed Army Institute of Research (WRAIR), Silver Spring, MD, and were performed in a facility accredited by the Association for the Assessment and Accreditation of Laboratory Animal Care International (AAALAC). The approved protocol was PO01-08: Systems biology studies to identify host indicators /therapeutic targets at early time periods post-exposure to *Y. pestis* (CO92) in African Green monkeys (*Chlorocebus aethiops*).

### Experiment details

The detailed experimental plan is described in our previous publication (Hammamieh et al. 2016). Briefly, the primates were individually housed in 3 × 2.6 × 2-ft. squeeze cages in free-standing enclosures equipped with standard enrichments and exposed to ambient environmental conditions inside an Animal Biosafety Level 3 (ABSL-3) containment laboratory, with a 12 h light/12 h dark cycle and temperatures ranging from 25 °C to 30 °C. The primates were fed a standard laboratory primate chow (TekLad, Madison, WI) with water provided *ad libitum* by Lixit valve (Lixit Corp., Napa, CA). The initial blood draws were performed 24 h before *Y. pestis* aerosol challenge and were the baseline samples for data analysis. The animals fasted for the last 6 h pre-challenge and were anesthetized using 4 mg/kg Telazol (Fort Dodge Animal Health, Fort Dodge, IA); after 15 min, they were aerosol challenged. All animals within each group were exposed on the same day at 30 min intervals, and subsequent blood draws were conducted at 6 h, 9 h, 12 h, 18 h, 24 h, 32 h and 42 h post-exposure.

### Bacterial inoculation and aerosol delivery

Detailed procedures were previously reported (Hammamieh et al. 2016). In short, suspension cultures of *Y. pestis* CO92 strain were prepared immediately prior to administration and anesthetized animals were inoculated using aerosol nebulizing generator. Purity of the aerosolized sample was assessed by colony morphology and growth on Congo Red-containing media.

### Blood collection and plasma separation

**Fig. 1:**
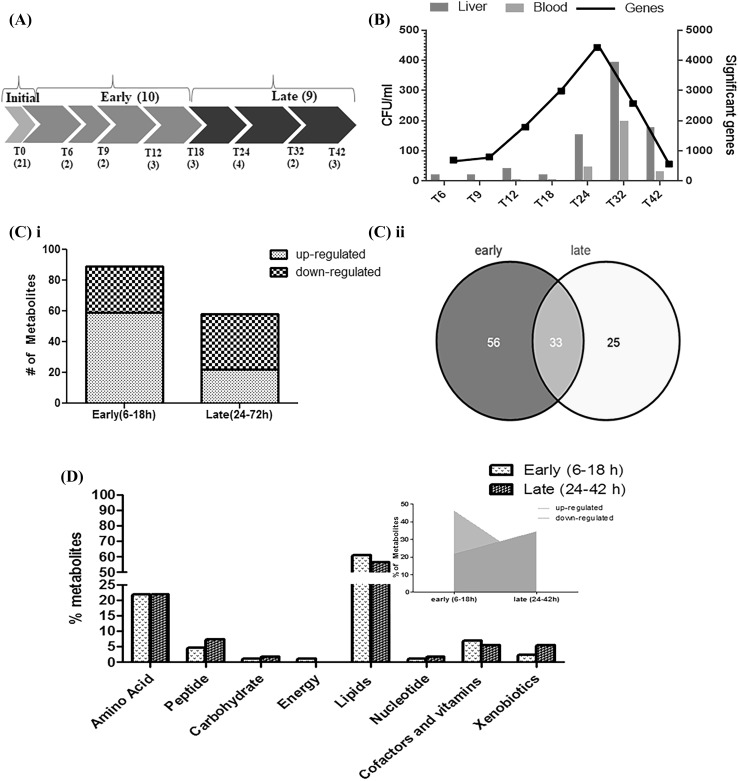
**a** *Experimental Plan*: We studied the longitudinal dynamics of plasma metabolites of the African Green monkey (*Chlorocebus aethiops*) infected by an aerosol exposure to *Y. pestis*. The baseline (T0 = initial time point) of the study was defined by the plasma samples drawn 24 h prior to exposing the monkeys to *Y. pestis*; the post-exposure blood samples were drawn at multiple time points, defined as T numbers in hours. The numbers of animals at each of the time points are shown within parentheses below the T numbers. The groupings of early and late time points are also shown. **b**. *Summarized bacterial load and gene expression data*: The data generated previously by (Hammamieh, Muhie et al. 2016) is summarized to show the bacterial load (in CFU) in blood and liver as well as significantly altered genes in blood at each of the time point post-exposure. **c** *Significant Metabolites*: The time points 6 h post-infection (p.i.) to 18 h p.i. are referred to as “early”, whereas the time points from 24 h to 42 h are considered “late.” The plasma samples collected before exposure were used as baseline for data analysis. (i) The number of metabolites at early and late time points. The graph is grouped by significantly elevated and reduced metabolites. (ii) The Venn diagram represents overlaps of significant metabolites at early and late time points. **d** *Super-pathways and Proportion of Metabolites*: Percentage of metabolites belonging to different super-pathways at early (6–18 h) and at late time points (24–42 h) with the proportion of metabolites being increased or decreased.. The trend for the lipid pathways at both time sets is emphasized (inset)

The sample collection schedule pre-exposure and at each time point is shown in Fig. 1a. The number of animals per time point varies from 2 to 4 and is described in Fig. 1a with a total 21 animals at pre-exposure time points. Venous blood was collected from each animal two times from the femoral vein with Vacutainer® CPT tubes (BD, Baltimore, MD) which were centrifuged at 1800 × *g* for 20 min at room temperature. Without disturbing the white cell layer, the clear plasma from the uppermost layer was transferred to a 15 mL tube and then stored at -80 °C. The plasma samples were filtered through a 0.2 micron filter, and an aliquot of each filtered sample was plated on Congo red agar plates in triplicate and incubated at 28 °C for 72 h to detect the presence of live bacteria in the samples. If no colonies were observed on the plates, samples were considered bacteria-free and removed from the BSL-3 facility. The resulting plasma from all the samples was bacteria-free and was used for metabolomics analysis. The no-infection control samples were also filtered in a similar way as infected samples using the micron filter as mentioned earlier.

### Plasma metabolomics

The metabolomics assays and analyses were conducted by Metabolon, Inc., (Morrisville, NC) at their facility. Untargeted metabolomic profiles of plasma samples were obtained using ultra-high performance liquid chromatography/tandem mass spectrometry (UHPLC/MS/MS) and gas chromatography/mass spectrometry (GC/MS) (Eckel-Mahan et al. 2012; Evans et al. 2009). The detailed procedure has been reported previously (Gautam et al. 2015).

Sample preparation was conducted using a series of organic and aqueous extractions to remove the protein fraction while allowing maximum recovery of small molecules. The resulting extract was divided into two fractions; one for gas chromatography (GC) analysis and second for liquid chromatography (LC) platforms. Samples were placed briefly on a TurboVap® (Zymark) to remove the organic solvent. Each sample was then frozen and dried under vacuum. Samples were then prepared for the appropriate instrument, either LC/MS or GC/MS.

The LC/MS portion of the platform was based on a Waters ACQUITY UPLC and a Thermo-Finnigan LTQ mass spectrometer, which consisted of an electrospray ionization (ESI) source and linear ion-trap (LIT) mass analyzer. The sample extract was split into two aliquots, dried, then reconstituted in acidic or basic LC-compatible solvents, each of which contained 11 or more injection standards at fixed concentrations. One aliquot was analyzed using acidic positive ion optimized conditions and the other using basic negative ion optimized conditions in two independent injections using separate dedicated columns. Extracts reconstituted in acidic conditions were gradient eluted using water and methanol both containing 0.1% Formic acid, while the basic extracts, which also used water/methanol, contained 6.5 mM Ammonium Bicarbonate.

The samples destined for GC/MS analysis were re-dried under vacuum desiccation for a minimum of 24 h prior to being derivatized under dried nitrogen using bistrimethyl-silyl-triflouroacetamide. The GC column was 5% phenyl and the temperature ramp was from 40 to 300 °C in a 16 min period. Samples were analyzed on a Thermo-Finnigan Trace DSQ fast-scanning single-quadrupole mass spectrometer using electron impact ionization. The instrument was tuned and calibrated for mass resolution and mass accuracy on a daily basis.

The LC/MS portion of the platform was based on a Waters ACQUITY UPLC and a Thermo-Finnigan LTQ-FT mass spectrometer, which had a linear ion-trap (LIT) front end and a Fourier transform ion cyclotron resonance (FT-ICR) mass spectrometer backend. For ions with counts greater than 2 million, an accurate mass measurement could be performed. Accurate mass measurements could be made on the parent ion as well as fragments. The typical mass error was less than 5 ppm. Ions with less than two million counts require a greater amount of effort to characterize. Fragmentation spectra (MS/MS) were typically generated in a data-dependent manner.

Peaks were identified using Metabolon’s proprietary software. Compounds were identified by comparison to library entries of purified standards or recurrent unknown entities. Identification of known chemical entities was based on comparison to metabolomic library entries of purified standards of more than 2000 commercially available purified standard compounds. The combination of chromatographic properties and mass spectra gave an indication of a match to the specific compound or an isobaric entity. Additional entities could be identified by virtue of their recurrent nature (both chromatographic and mass spectral). Metabolon data analysts used proprietary visualization and interpretation software to confirm the consistency of peak identification among the various samples. Library matches for each compound were checked for each sample and corrected if necessary. For all analyses, missing values (if any) were imputed with the observed minimum for that particular compound. The statistical analyses were performed on natural log-transformed data to reduce the effect of any potential outliers in the data.

Some mass and chromatographic peaks were observed repeatedly, but are currently unidentified. Each known metabolite is annotated with a superpathway corresponding to its general metabolic class, and a subpathway representing more specific metabolic pathways from information available in public databases (e.g., KEGG (Kyoto Encyclopedia of Genes and Genomes), HMDB (Human Metabolome Database) and text books) and Metabolon’s internal knowledge base. These superpathways and subpathways were constructed prior to the statistical analyses.

### Plasma proteins

An aliquot of each sample was shipped to Rules-Based Medicine, Inc. (Austin, TX) for protein analysis. All samples were stored at − 80 °C until tested and were analyzed for Human Discovery Map v.1.0 Antigens (Myriad-RBM) using Luminex technology.

### Data analysis

Data was expressed as the mean ± standard deviation (SD) for each group. The significance of differences between groups was assessed by the student’s *t*-test. Differences were considered significant at *p* < 0.05. Continuous variables measured at several time points were analyzed with traditional ANOVA (analysis of variance) methods for a repeated measures design, followed by one or more multiple comparison procedures to compare baseline values with the other time points. Lastly, all confidence intervals were performed at the 95% level of significance and all statistical tests were performed at the 0.05 alpha level. The column statistical analysis and figures were generated using GraphPad Prism v5.

### Enrichment analysis

Pathway enrichment analysis was done using Metabolync software (Metabolon, Inc.) for each individual pair-wise comparison. This pathway enrichment displayed the number of experimentally regulated compounds relative to all detected compounds in a pathway, and compared it to the total number of experimentally regulated compounds relative to all detected compounds in the study. A pathway enrichment value greater than one indicates that the pathway contains more experimentally regulated compounds relative to the study overall, suggesting that the pathway may be a target of the experimental perturbation. Enrichment was defined as (k/m)/(n/N), where k is the number of significant metabolites in a pathway, m is the total number of detected metabolites in the pathway, n is the total number of significant metabolites, and N is the total number of detected metabolites. Enrichment analysis of the relevant pathways and networks was performed using MetaboAnalyst 3.0, a web-based tool that combines results from a powerful pathway enrichment analysis concerning the conditions under study (Xia and Wishart 2016). MetaboAnalyst’s directed graph function uses the high-quality KEGG (http://www.genome.jp/kegg/) pathway database as its backend knowledge base. The analysis used fold change data following univariate analysis function (hypergeometric test over-representation analysis and relative-betweenness centrality pathway topology analysis).

### Network analysis

To further understand the biological significance of differentially expressed plasma metabolites and proteins, Ingenuity Pathway Analysis (IPA) (Qiagen, Inc., Hilden, Germany) was used to analyze canonical pathways and relationships in the data. Disease and functional protein networks were combined with upstream regulator analyses of differentially expressed metabolites, and a resulting Z-score of ≥ 2 or ≤ − 2 was considered to indicate significant activation or inhibition, respectively.

### Integrative gene and metabolite analysis

NHP metabolite-gene interaction networks across time points (6 h–42 h) were analyzed and constructed using MetScape v3.1.2 (http://www.cytoscape.org) (Karnovsky et al. 2012), and visualized using gephi v0.9.1 (https://gephi.org/). Interaction networks were built from differentially regulated transcripts (Hammamieh et al. 2016) and differentially altered metabolites.

### Cluster analysis

A Manhattan distance method was used to identify subpathway clusters in regard to whether metabolites increased or decreased vs. their matched baseline controls. Each metabolite of a subpathway was assigned either a 0 or a 1 for having its mean plasma level higher or lower in each comparison. Here, the actual direction of regulation is not important, only the pattern relative to other metabolites in other pathways. We reason that pathways with similar patterns of metabolite co-regulation are likely to be physiologically related. To find co-regulation relationships among pathways, we computed the average distance of metabolites in a subpathway, and each metabolite of a subpathway was compared pairwise with every other metabolite of the pathway. Two metabolites with the same 1’s and 0’s in all eight comparisons (same pattern of regulation across experimental conditions) have a distance of zero. Two metabolites with different 1’s and 0’s in all eight comparisons have a distance of 8. The total up vs. down Manhattan distance across all pairs of metabolites within a subpathway was tested against the null distribution of analogous distances created by taking 10,000 random samplings from all the metabolites of the number in the subpathway of interest. An approximate (slightly conservative) *p* value was then calculated as (b + 1)/(m + 1), where b is the number of sample distances greater than the actual subpathway distance and m is the number of random samplings (10,000), as described in (Phipson and Smyth 2010).

## Results and discussion

### Changes in metabolites and superpathways

The goal of this study was to profile the metabolic changes in the plasma associated with the host response to *Y. pestis* infection in NHPs. We infected African Green monkeys with aerosolized CO92 strain of *Y. pestis*, and isolated plasma samples for metabolomics profiling at 6 h, 9 h, 12 h, 18 h, 24 h, 32 h and 42 h after infection. We dropped the samples post 42 h because of animal lethality observed at later time points post-exposure leading to inadequate sample size. As reported previously (Hammamieh et al. 2016) the presence of bacteria in blood was reported in one of three animals as early as 9 h after exposure whereas liver showed an early and consistent bacterial load at 6 h (Fig. 1b). The significant number of genes from each of the times post-exposure is also summarized in Fig. 1b. Minimal changes in the body temperature were observed in these animals and most of the temperature differences were attributed to the individual variances as reported earlier (Hammamieh et al. 2016). The number of animals at each time point is listed in Fig. 1a. A total of 384 metabolites were detected, 247 of which were identified compounds and the remaining 137 were unidentified peaks (Table S1). The transcriptome data published earlier grouped 45 min to 18 h as the early time point group and 24–42 h as the late time point group, which was justified by the bell-shaped transcriptome expression profile (Hammamieh et al. 2016). The 45 min datapoint was missing from the current study and rest of the groupings were kept uniform across the study. The early time point samples were those taken from 6 h to 18 h p.i., whereas the late time point set consisted of the 24 h– 42 h samples which helped us to enhance the statistical power of the analysis. The data from the pre-infection time point was used as the baseline sample for analysis. Early time points (6 h–18 h) showed a greater number of significantly-changed metabolites (Fig. 1c), with 30 metabolites at decreased levels and 59 at increased levels. At the late time points (24 h–42 h), 36 metabolites were at lower level and 22 were at higher level (Table S2). The early time points had 56 unique metabolites, and the late time points had 25 unique metabolites. 33 metabolites were common among early and late time points (Fig. 1c). The maximum number of significant metabolites peaked at the 12 h time point (data not shown), much earlier than the peak bacterial load that was observed at the 24 h time point (Hammamieh et al. 2016). This was also a little earlier than the peak of changes in transcriptomic data observed at 18 h–24 h in our previous study (Hammamieh et al. 2016). Of these significantly altered compounds, ~ 59% belonged to the lipid class including lysolipids, bile acids, sterols, medium /long chain fatty acids, other fatty acids and inositol metabolites. Of the metabolites, 24% represented amino acids, 5% peptides, 2% carbohydrates, 1% energy compounds, and the rest were other classes (Fig. 1d). The lipid superpathway had similar proportions of metabolites at both early and late time points; however, the number of elevated metabolites was higher at early time points (46%) as compared to late time points (22%). The proportion of metabolites with reduced levels increased from 15 to 35% from early to late time points (Fig. 1d). After excluding metabolites with missing values, 45 of 230 known metabolites were lower at late time points (*p* ≤ 0.1, mean difference of 0.2 or greater). Of these, 30 were lipids (of a total of 108 lipids) with an enrichment (hypergeometric test) at *p* < 0.001. Using the same criteria, six metabolites were increased at late time points, and four of these were amino acid metabolism (out of 58 total from amino acids metabolism) with an enrichment at *p* < 0.001. The cluster analysis showed that energy- and lipid-related metabolites were clustered close to each other at all time points p.i. (Fig. S1).

### Altered lipid superpathway related metabolites

**Fig. 2:**
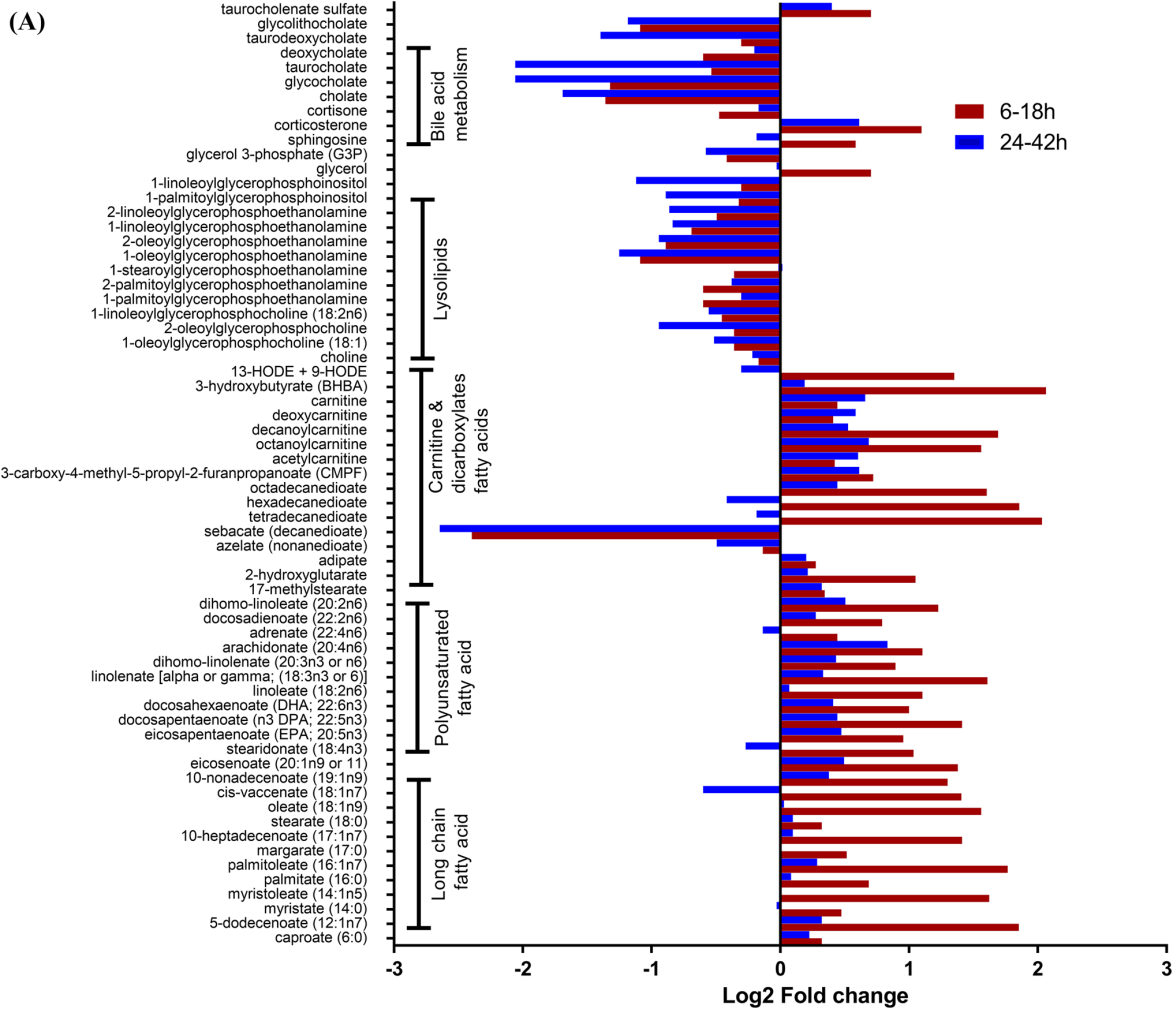

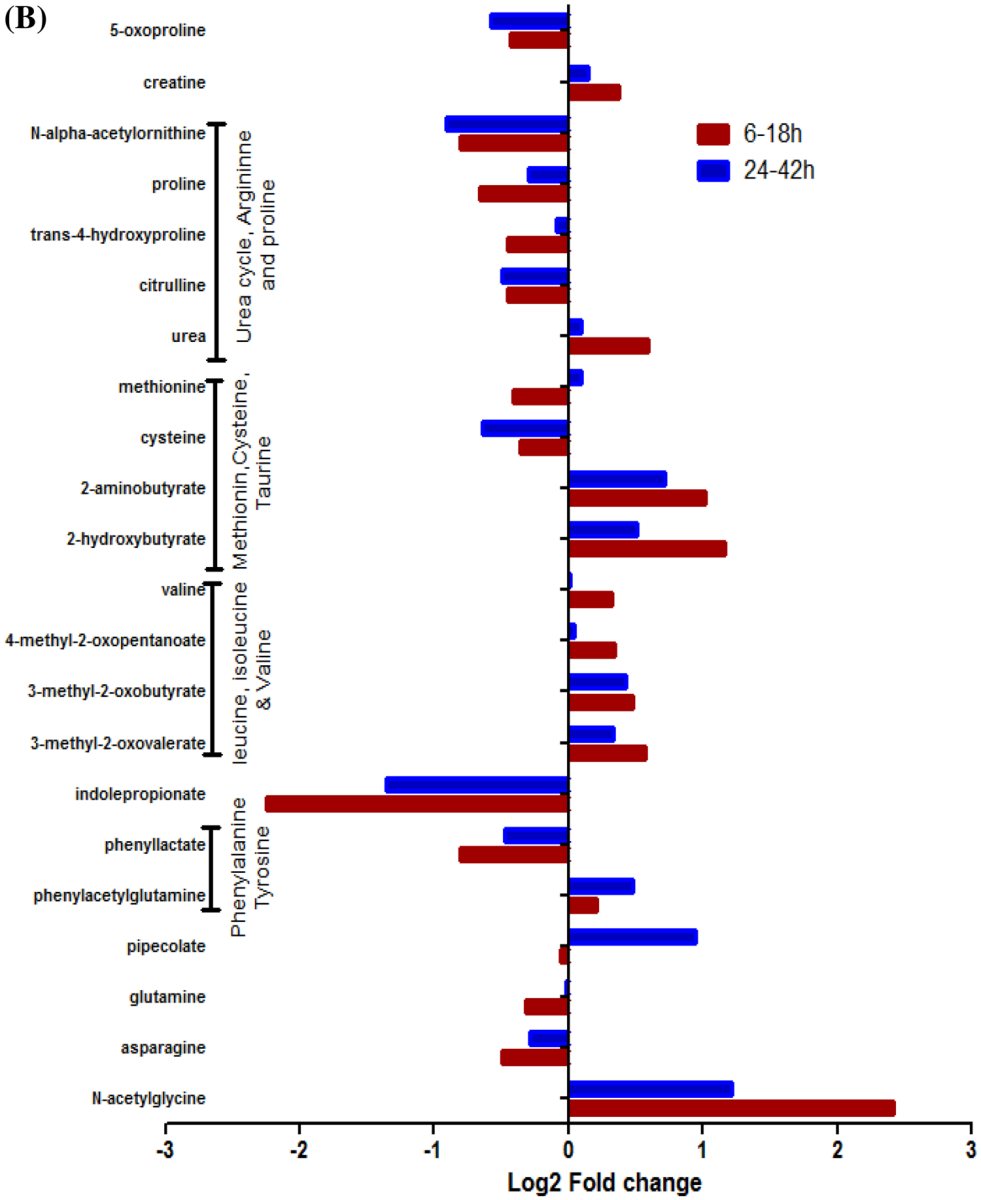
**a** *Metabolites in the lipid superpathway*: Log2 fold change of significantly (*p* < 0.05) identified metabolites in lipid pathways that are sub-grouped. **b** *Metabolites in the amino acid superpathway*: Log2 fold change of significantly (*p* < 0.05) identified metabolites in amino acid pathways that are sub-grouped

We observed several classes of lipids that changed throughout the course of infection. The long chain fatty acids, polyunsaturated and dicarboxylate fatty acids, and carnitines were significantly higher at early time points (Fig. 2a). The metabolites from lysolipids and bile acids metabolism pathways were downregulated, and significant values were observed at late time points. Fatty acids and lysolipids are incorporated into membranes and may exert an effect on the membrane permeability, morphology and stability. Subsequent dysregulation may lead to increased barrier permeability (Puertollano and Álvarez de Cienfuegos 2002). The fatty acids have also been reported to be modulators of immune function (Fritsche 2006; Puertollano et al. 2002). It has been hypothesized that these changes may alter membrane fluidity, lipid peroxide formation, and eicosanoid production (Puertollano and Álvarez de Cienfuegos 2002). Carnitines, which are involved in transporting long chain fatty acids across the mitochondrial membrane, were higher immediately after exposure. Several medium and short chain carnitines, including octanoylcarnitine, decanoylcarnitine and acetylcarnitine, were significantly increased at various time points during the infection (Fig. 2a). These changes suggest increased fatty acid β-oxidation and mitochondrial flux. The changes in bile acid metabolism can affect lipid absorption (Staels and Fonseca 2009) and, therefore, may have led to the changes in free fatty acids and triglycerides that were reflected in the plasma metabolites. Bile acid synthesis occurs exclusively in liver, and accumulation of toxic bile acid metabolites may cause inflammation, apoptosis and cell death (Chiang 2013). The presence of bile acids metabolites in the plasma along with bacterial load in liver (Hammamieh et al. 2016) underscores the prognostic importance of liver dysfunction after infection. Recently, in vivo imaging suggested that secondary lymphoid tissues such as lymph nodes, liver and spleen are the main sites of bacterial multiplication when bacterial tracking was studied (Nham et al. 2012). Although, the study tracked bubonic plague, it gave a snapshot that bacteria have extraordinary capacity to disseminate, which may lead to rapid fatality of the host. The progression to lethal sepsis with augmented liver injury has been reported for pneumonic plague and is encoded by factors on pCD1 plasmid (Doyle et al. 2010). In our study, a significant increase in essential and long-chain fatty acids after infection with lipolysis of triglycerides, fatty acid mobilization, and subsequent β-oxidation of these free fatty acids indicated a need for increased energy.

### Altered amino acid superpathways

A minor fraction of branched chain amino acids (BCAA) are metabolized mainly in the liver and the rest are transported to sites of metabolism by the systemic circulation (De Simone et al. 2013). Under conditions of increased energy demand, the levels of branched chain amino acids (BCAA) and keto acids (BCKA) in the plasma are elevated (Harper et al. 1984). The BCAA valine, BCKAs 3-methyl-2-oxobutyrate (alpha-ketoisocaproate) and 3-methyl-2-oxovalerate (alpha-keto-beta-methylvalerate), and downstream metabolite 3-hydroxy-isobutyrate were elevated at various times during the infection (Fig. 2b). These changes, together with the previously described alterations in lipid metabolism, indicated that an overall increased energy demand was placed on the host following infection. We also observed elevation of the ketone bodies acetoacetate and 3-hydroxybutyrate that could have resulted from an excess of acetyl-CoA or from the catabolism of certain ketogenic amino acids (leucine, isoleucine, lysine, phenylalanine, tyrosine and tryptophan). The rate of ketogenesis is coupled to the supply of fatty acids and the regulation of β-oxidation (Salway 2004), thus the production of ketone bodies in the blood has been used as a marker for the rate of fatty acid β-oxidation. 3-hydroxybutyrate was significantly increased at early hours p.i., again indicating that there was an early increase in the rate of β-oxidation from the mobilization of fatty acids.

### Pathway enrichment analysis

The pathway enrichment analysis using Metabolync revealed significant perturbations of 11 pathways that were upregulated for the entire time period of infection, including, but not limited to, metabolism of fatty acids (dicarboxylates); glycerolipids; polysaturated fatty acids; primary bile acids; steroids; ascorbate and aldarate; methionine, cysteine and taurine (Table 1). A significant accumulation of dicarboxylic fatty acids, in particular, tetradecanedioate, hexadecanedioate and octadecanedioate, at early time points suggests there was increased ω-oxidation in the smooth endoplasmic reticulum in addition to β-oxidation in the mitochondria of the infected monkeys. Under normal physiological conditions, ω-oxidation of fatty acids is a minor pathway that accounts for a small fraction of the total fatty acid oxidation in the liver. 5-oxoproline, an intermediate in glutathione metabolism, was found to be affected after infection. Glutathione level is known to be altered in many inflammatory conditions and known to be lower in animal models during the initial phase of septic shock, and its turnover is increased during acute phase of sepsis in animal model (Malmezat et al. 2000). The antioxidant potential of glutathione can play a role in immunity and can act as a signaling molecule (Ghezzi 2011). It has been postulated that glutathione may not be just an inhibitor of inflammation, but may also regulate innate immunity that could be favorable to the host (Ghezzi 2011). However, recent evidence suggests that *Y. pestis* utilizes glutathione in host tissues as a virulence strategy to quicken the plague pathogenesis (Mitchell et al. 2017). In this study, the cap protein of the bacterial type III secretion needles (LcrV) is modified by host glutathione leading to the high virulence of *Y. pestis* in rodents in the case of bubonic plague. Vitamin B6 metabolism was enriched during the entire time course of the study groups where pyridoxate, an important precursor, was observed to be lower after infection. The B6 vitamins are primarily metabolized in liver (Merrill and Henderson 1990), and a lower level in plasma samples have been reported in patients with decompensated cirrhosis or subacute hepatic necrosis (1977). Further, while B vitamins are not synthesized by humans, these can be synthesized by bacteria, so it is not surprising to observe vitamin B6 metabolism after *Y. pestis* exposure. Many amino acid pathways, such as metabolism of ketone bodies, creatinine, and oxidative phosphorylation, were mainly disrupted at early time points. In contrast, there were some pathways found to be enriched only at late time points (Table 1). The changes in primary bile acids may have led to changes in the levels of secondary bile conjugates (Staels and Fonseca 2009). The changes in bile acid pathways can have an impact on the liver, and the functional analysis using genes of interest after infection resulted in the annotation of liver failure as a key pathway with the bacterial load increasing in the tissues as early as 6 h after infection (Hammamieh et al. 2016). The most noticeable feature observed was an increase in plasma free-fatty acid metabolism at multiple time points. This increase could be a result of higher lipolysis or the breakdown of membrane lipids in adipose tissue and liver.

**Table 1:** Enriched pathways during the course of infection

Enriched pathways	T6_18H	T24_T42H
Glutathione metabolism	2.85	4.51
Polypeptide	2.85	4.51
Vitamin B6 metabolism	2.85	4.51
Fatty acid metabolism (acyl carnitine)	2.85	3.01
Polysaturated fatty acid (n3 and n6)	2.85	2.46
Glycerolipid metabolism	2.85	2.25
Fatty acid (dicarboxylate)	2.49	1.69
Methionine cysteine SAM and taurine	2.14	3.38
Primary bile acid metabolism	1.9	4.51
Steriod	1.43	1.13
Ascorbate and aldarate metabolism	1.3	2.25
Ketone bodies	2.85	
Pantothenate and CoA metabolism	2.85	
Long chain fatty acid	2.61	
Alanine and aspartate metabolism	1.43	
Creatinine metabolism	1.43	
Hemoglobin and porphyrin metabolism	1.43	
Oxidative phosphorylation	1.43	
Urea cycle arginine and proline metabolism	1.43	
Leucine, isoleucine and proline metabolism	1.04	
Acetylated peptides		4.51
Phospholipid metabolism		4.51
Benzoate metabolism		3.01
Carnitine metabolism		3.01
Lysine metabolism		2.25
Gamma-glutamyl amino acid		1.93
Tocopherol metabolism		1.5
Lysolipid		1.45
Secondary bile acid metabolism		1.13

The analysis is done using Metabolync, where pathways unique at early and late time points of infection are also listed

**Fig. 3:**
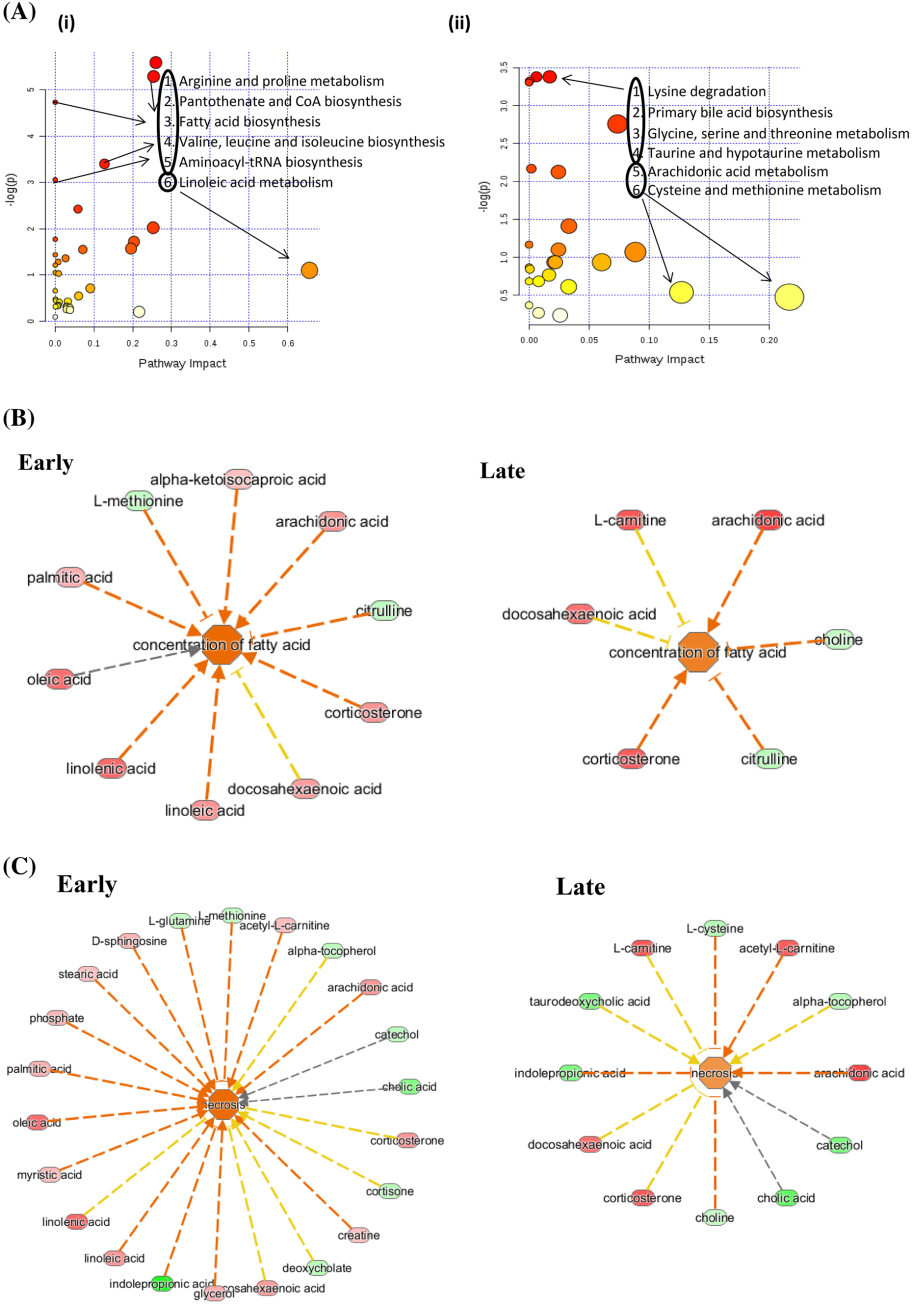
**a** MetaboAnalyst analysis: Enrichment analysis showing metabolite map to multiple biosynthetic pathways at (i) early time points (6–18 h) and at (ii) late time points (24–42 h). Metabolites are plotted according to the Global Test *p*-value (vertical axis, intensity of color) and impact factor (horizontal axis, size of circle). **b** Concentration of fatty acids: One of the top regulated network as identified by IPA at early (6–18 h) and late time points (24–42 h). **c** Necrosis: One of the top regulated network as identified by IPA at early (6–18 h) and late time points (24–42 h)

Using MetaboAnalyst to identify biologically meaningful patterns enriched in the data also guided us to similar results as observed earlier. Here, potential target metabolic pathway analysis uses impact-value ≥ 0.10 to identify the pathways that are important for the host response to infection. These metabolites are also responsible for the metabolism of linoleic acid, arachidonic acid, cysteine and methionine (Fig. 3a and Table S3). Arginine and proline metabolism; the biosynthesis of aminoacyl-tRNA; pantothenate and CoA; fatty acids and valine, leucine and isoleucine were found to be disturbed at early time points. The top metabolic pathways of importance at late time points were lysine degradation; primary bile acid biosynthesis; glycine, serine, and threonine metabolism and taurine and hypotaurine metabolism.

We identified arachidonic acid metabolism as a high impact pathway (Fig. 3a) at late time points of infection, and derivatives from arachidonic acid metabolism are influential mediators of inflammation. Eicosanoids are powerful lipid mediators derived from arachidonic acid, and they are known to regulate many levels of inflammation. The role of lipids in infection is not a widely studied area, but there have been reports that leukotriene generation was suppressed in the presence of intracellular bacteria (Gröne et al. 1992). Also, some eicosanoids are suspected to act as molecular sensors for neutrophils recruitment and others may act by regulating bacterial uptake (Tyrkalska et al. 2016).

The metabolite cysteine is part of a highly impacted pathway at late time points. Cysteine is a sulfur-containing amino acid that plays a critical role in protein structure by forming disulfide bonds with other cysteine residues (Brosnan and Brosnan 2006). The exact mechanism whereby cysteine may have played a role in this study remains unclear, but this amino acid is known to be involved in glutathione biosynthesis, which leads to changes in an important cap protein of the bacterial type III secretion system. Subsequently, these protein changes lead to the high virulence of *Y. pestis* in the mouse and rat models which manifests as bubonic plague (Mitchell et al. 2017). It is known that peptide-conjugated lipid inflammatory mediators such as cysteinyl leukotrienes along with glutathione or glutathione cleavage products may have a key role in inflammation (Fanning and Boyce 2015).

### Ingenuity pathway analysis (IPA)

Based on the number of pathways observed, molecular and cellular functions were significantly enhanced at early time points as compared to late time points. The only canonical pathway that passed the Benjamini-Hochberg (BH) correction was the citrulline biosynthesis pathway of which five of the 18 metabolites were affected. Citrulline, glutamine and proline were all reduced, while phosphate and urea were elevated. Citrulline biosynthesis occurs mainly in the intestine, and citrulline released from the intestine is metabolized by the kidneys (Windmueller and Spaeth 1981). It has been reported that impairment of citrulline metabolism is linked to kidney failure, and the citrulline pathway was significantly changed at an early time point of infection. The most significantly enriched molecular and cellular functions that were observed at all time points are shown in Table 2. Carbohydrate metabolism was the top category that was highly significant, and the molecules in this category involved at early time points were oleic acid, creatine, d-mannose, l-methionine, hexanoic acid, palmitic acid, myristic acid, glycerol, phosphate, linoleic acid, and stearic acid. Citrulline, choline, corticosterone, and taurocholic acid were significantly enriched at late time points. A recent study determined that *Y. pestis* requires carbohydrate metabolism during colonization in the host (Pradel et al. 2014), and this has been studied in regards to bubonic infection. Also, since the infection is temperature-dependent, it has been found that different types of carbohydrates are metabolized after this temperature transition (Heroven and Dersch 2014). Energy metabolism was significantly enriched at early time points along with four other categories; protein degradation, DNA replication, recombination and repair, and gene expression. However, whether this finding reflects the high energy expenditure that is required to fight the infection or a general inhibition of carbohydrate metabolism as a result of the infection remains unanswered.

**Table 2:** Disease and biofunctions for all categories with significant − log (BH) *p*-value

Category	Early(6–18 h)	late (24–42 h)
Carbohydrate metabolism	6.04E− 05–1.14E−01	9.86E− 02–1.19E−01
Energy production	6.04E−05–1.22E−01	
Small molecule biochemistry	6.04E−05–1.34E−01	3.41E−02–1.3E−01
Cell cycle	8.32E−04–1.08E−01	9.86E−02–1.19E–01
Cell signaling	8.32E−04–1.08E−01	7.55E−02–9.86E−02
Molecular transport	8.32E−04–1.34E−01	3.41E− 02–1.3E− 01
Vitamin and mineral metabolism	8.32E−04–1.14E−01	1.3E−01–1.3E−01
Lipid metabolism	8.32E−04–1.34E−01	3.41E−02–1.3E−01
Developmental disorder	1.42E−03–1.14E−01	3.41E−02–1.19E−01
Gastrointestinal disease	1.42E−03–1.34E−01	3.41E−02–1.19E−01
Hepatic system disease	1.42E−03–1.34E−01	3.41E−2–1.19E−01
Organismal injury and abnormalities	1.42E−03–1.34E−01	3.41E−02–1.36E−01
Cell death and survival	3.62E−03–1.34E−01	9.86E−02–1.36E−01
Cancer	4.37E−0 3–1.34E−01	9.86E−02–1.35E−01
Cellular assembly and organization	5.07E−03–1.34E−01	9.86E−02–1.19E−01
DNA replication, recombination, and repair	8.4E−03–1.08E−01	
Nucleic acid metabolism	8.4E−0 3–1.08E−01	7.55E−02–9.86E− 02
Cellular development	1E−0 2–1.34E− 01	9.86E−02–1.26E− 01
Cellular growth and proliferation	1E−02–1.34E− 01	9.86E−02–1.26E− 01
Protein synthesis	1.1E− 02–1.31E− 01	
Cellular compromise	1.1E−0 2–1.14E−01	7.74E−03–1.19E−01
Organismal survival	1.1E− 02–1.1E− 02	1.19E−01–1.21E−01
Free radical scavenging	1.4E− 02–1.34E−01	7.55E−02–1.3E−01
Tumor morphology	1.4E−02–1.34E−01	9.86E−02–1.19E−01
Endocrine system development and function	1.4E−02–1.08E−01	9.86E−02–1.3E−01
Hematological system development and function	1.4E−02–1.34E−01	9.86E−02–1.36E−01
Hepatic system development and function	1.4E−02–1.08E−01	9.86E−02–9.86E−02
Humoral immune response	1.4E−02–1.4E−02	
Lymphoid tissue structure and development	1.4E−0 2–1.08E−01	1.19E −01–1.19E−01
Tissue morphology	1.4E−02–1.14E−01	9.86E−02–1.19E−01
Behavior	2.67E−02–1.13E−01	9.86E−02–1.19E−01
Endocrine system disorders	2.67E−02–1.14E−01	9.86E−02–9.86E−02
Metabolic disease	2.67E−02–1.14E−01	3.41E−02–1.3E−01
Skeletal and muscular disorders	2.67E− 02–1.14E−01	9.86E−02–9.86E−02
Nervous system development and function	3.73E−02–1.13E − 01	9.86E−02–1.3E−01
Neurological disease	3.73E−02–1.22E−01	3.41E−02–1.36E−01
Cellular function and maintenance	4.21E−02–1.34E−01	9.86E−02–1.19E−01
Hematopoiesis	4.94E−02–1.14E−01	9.86E−02–1.19E−01
Tissue development	4.94E−02–1.14E−01	9.86E−02–1.19E−01
Cell morphology	5.02E−02–1.34E −01	9.86E − 02–1.19E − 01
Cell-To-Cell signaling and interaction	5.02E− 02–1.34E−01	9.86E−02–1.36E−01
Immune cell trafficking	5.02E−02–1.34E−01	9.86E−02–1.36E−01
Inflammatory response	5.02E−02–1.34E−01	9.86E−02–1.36E−01
Connective tissue development and function	5.48E−02–1.14E−01	9.86E−02–1.19E−01
Cardiovascular disease	6.44E− 02–1.22E−01	9.86E−02–1.19E−01
Drug metabolism	6.44E−02–1.08E−01	9.86E−02–1.3E−01
Organ morphology	7.07E− 02–1.22E−01	9.86E−02–9.86E−02
Skeletal and muscular system development and function	7.07E−02–1.34E −01	1.19E−01–1.19E−01
Hair and skin development and function	7.07E− 02–7.07E−02	
Reproductive system development and function	7.07E−02–1.08E−01	9.86E−02–9.86E−02
Digestive system development and function	8.81E− 02–1.08E−01	9.86E−02–1.19E−01
Organ development	8.81E− 02–1.08E−01	9.86E−02–1.19E−01
Amino acid metabolism	9.97E−02–1.08E−01	9.86E−02–1.19E−01
Respiratory disease	9.97E−02–1.08E−01	9.86E−02–1.19E−01
Dermatological diseases and conditions	1E−01–1.08E−01	9.86E−02–1.19E −01
Inflammatory disease	1E−01–1.08E−01	3.41E−02–1.36E−01
Renal and urological disease	1.08E−01–1.31E−01	9.86E−02–1.19E−01
Cellular movement	1.08E−01–1.14E−01	9.86E−02–1.19E−01
Embryonic development	1.08E−01–1.08E−01	9.86E−02–1.3E−01
Hematological disease	1.08E−01–1.08E−01	9.86E−02–1.3E−01
Immunological disease	1.08E−01–1.08E−01	9.86E−02–1.36E−01
Organismal development	1.08E−01–1.22E −01	9.86E−02–1.3E−01
Auditory and vestibular system development and function	1.08E−01–1.08E−01	9.86E− 02–9.86E−02
Cardiovascular system development and function	1.08E−01–1.22E−01	9.86E− 02–1.19E −01
Connective tissue disorders	1.08E−01–1.08E−01	9.86E−02–9.86E−02
Gene expression	1.08E−01–1.16E −01	9.86E − 02–1.19E−01
Hereditary disorder	1.08E− 01–1.14E −01	3.41E−02–1.19E−01
Infectious diseases	1.08E−01–1.08E −01	3.41E−02–1.19E −01
Nutritional disease	1.08E− 01–1.13E −01	4.25E−02–4.25E−02
Ophthalmic disease	1.08E− 01–1.08E −01	
Organismal functions	1.08E−01–1.08E−01	
Post-Translational Modification	1.08E−01–1.08E−01	1.19E−01–1.19E−01
Protein degradation	1.08E−01–1.31E−01	
Protein trafficking	1.08E−01–1.08E−01	1.19E− 01–1.19E−01
Psychological disorders	1.08E− 01–1.14E−01	9.86E −02–9.86E−02
Respiratory system development and function	1.08E−01–1.08E−01	9.86E− 02–9.86E − 02
Cell-mediated immune response		1.19E− 01–1.19E− 01
Hypersensitivity response		1.19E − 01–1.19E −01
Renal and urological system development and function		1.19E− 01–1.19E−01
Reproductive system disease		9.86E−02–1.19E−01

The significant toxicity (TOX) functions that were defined by IPA were significantly increased levels of alanine aminotransferase (ALT) and liver necrosis at early time points, and liver cholestasis at late time points of infection. The changes in the ALT level are an integral part of the evaluations of patients with liver disease (Kim et al. 2008), and our data suggested that the liver was immediately affected. With the onset of cholestasis, the disruption of bile flow occurs in hepatocytes, and this was exactly what we observed in our enrichment analysis. There were many fatty acids metabolites in the present dataset, where deoxycholate, oleic acid, palmitic acid, cholic acid, arachidonic acid, stearic acid and linoleic acid were observed at early timepoints whereas taurodeoxycholic acid, cholic acid, taurocholic acid and glycocholic acid were observed at late time points. *Y. pestis* is known to utilize palmitic acid as a carbon and energy source (Moncla et al. 1983) and has enzymes for fatty acid degradation. We studied interaction networks among differentially expressed metabolites, which reveals the interactions between diseases and functions. A thorough investigation of the TOX functions is summarized in Table 3. The concentration of fatty acids and necrosis were the top regulated functions that were activated, and the differences between these networks at early and late time points are shown in Fig. 3b, c. At early time points of infection, ten metabolites were involved in the fatty acids network and were highly activated, as compared to six metabolites at late times of infection (Fig. 3b). The mammalian host provides a fatty acid rich environment and may be enhancing the virulence of the bacteria (Moncla et al. 1983). *Y. pestis* survives in macrophages during its early invasion process and develops resistance to phagocytosis. The release of *Y. pestis* from macrophages is associated with necrosis and/or apoptosis (Ke et al. 2013). In this study, we observed that metabolites were higher for necrosis at early times of infection, thereby indicating immediate activity as compared to the late time points (Fig. 3c).

**Table 3:** Disease and biofunctions with activation Z score

Diseases and bio functions	Early (6–18 h)	Late (24–42 h)
Concentration of fatty acid	2.76	1.44
Necrosis	2.45	1.06
Non-melanoma solid tumor	− 2.17	− 1.00
Quantity of Ca2+	3.05	0.00
Organismal death	− 0.71	− 2.01
Accumulation of triacylglycerol	1.61	− 1.07
Accumulation of lipid	2.25	− 0.37
Growth of tumor	− 2.03	− 0.47
Production of reactive oxygen species	1.46	1.01
Apoptosis of tumor cell lines	2.47	0.00
Synthesis of cyclic AMP	0.00	− 2.45
Oxidation of glucose-6-phosphate	− 2.45	0.00
Cell viability of tumor cell lines	− 1.54	− 0.90
Cell death of tumor cell lines	2.39	0.00
Oxidation of monosaccharide	− 2.35	0.00
Concentration of eicosanoid	2.33	0.00
Apoptosis of pancreatic cancer cell lines	2.18	0.00
Cancer	− 1.62	− 0.55
Apoptosis of endothelial cells	2.17	0.00
Accumulation of acylglycerol	2.06	0.00
Quantity of reactive oxygen species	1.14	0.88
Synthesis of fatty acid	1.11	0.90
Synthesis of nitric oxide	− 0.10	− 1.90
Proliferation of CD4 + T-lymphocytes	2.00	0.00
Apoptosis of vascular endothelial cells	2.00	0.00

### Proteomics analysis

**Fig. 4:**
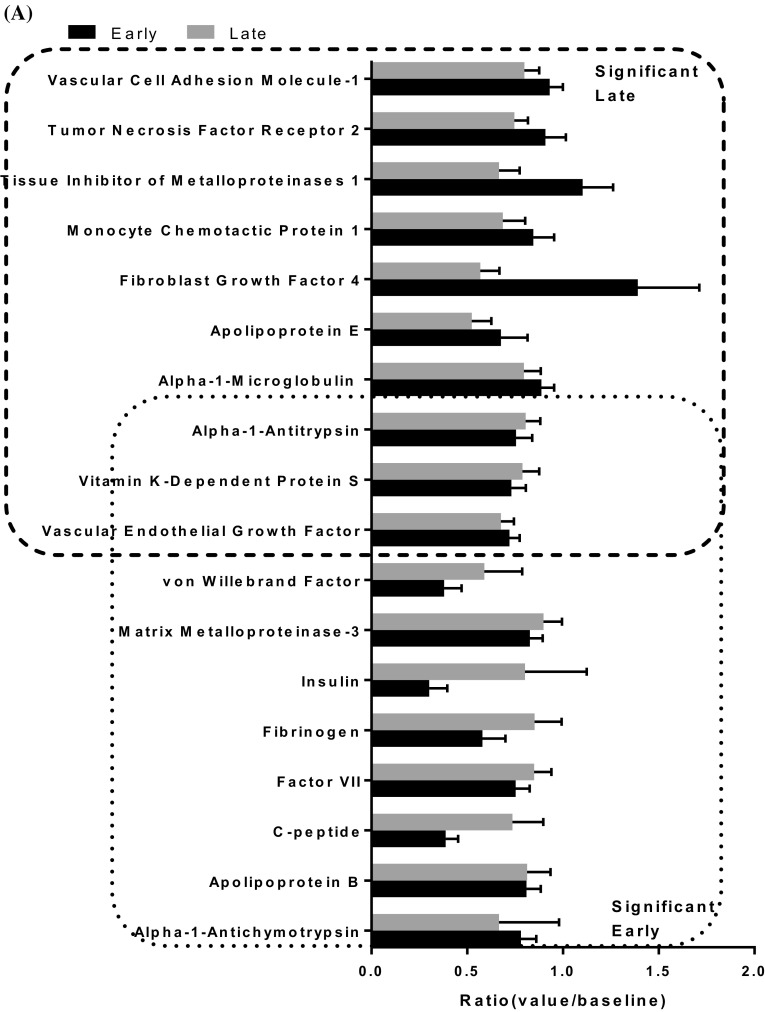

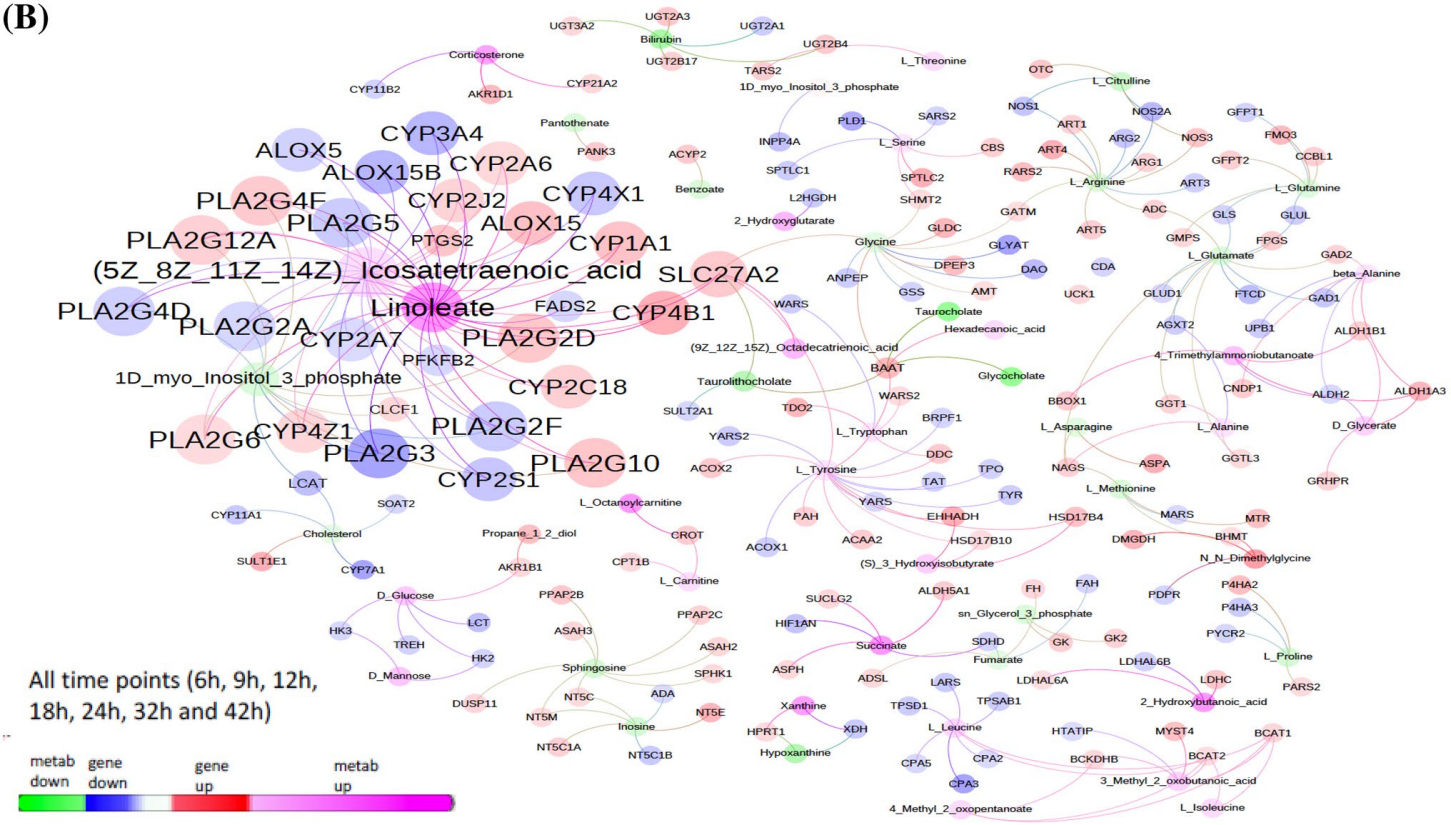
**a** Protein assay: Ratio of significantly (*p* < 0.05) identified proteins are sub-grouped as early and late groups. The common proteins between the two groups is shown in overlapping regions. **b** Integrative gene-metabolite network: Significant genes identified from blood samples in previously published manuscript (Hammamieh et al. 2016) and metabolites were combined from all of the time points for integrated analysis. The larger node size reflects the modularity. Interaction networks were constituted from differentially regulated transcripts (blue colored nodes = down-regulated transcripts; red colored nodes = up-regulated transcripts) and differentially altered metabolites (green nodes = decreased metabolites; magenta colored nodes = elevated metabolites)

We identified 12 proteins that were significant (*p* value < 0.05) at early time point and 11 proteins that were significant (*p* value < 0.05) at late time points. Three of these proteins including vascular endothelial growth factor, vitamin K-dependent protein S and Alpha-1-antitrypsin were common for both of the time groups (Fig. 4a). Pathway analysis using IPA showed coagulation system as top canonical pathway with protein S and von Willebrand factor proteins involved in the pathway. The vitamin K plasma protein functions as a cofactor for anti-coagulant protease, which activated protein C to inhibit blood coagulation. The von Willebrand factor is a glycoprotein involved in hemostasis and is known to be involved in the adhesion of platelets to sites of vascular injury and the transport of various proteins in the blood. The proteins significant at late time point showed hepatic fibrosis as the top canonical function. Here the proteins that were known to be involved are C-C motif chemokine ligand 2, TIMP metallopeptidase inhibitor 1, TNF receptor superfamily member 1B, vascular cell adhesion molecule 1 and vascular endothelial growth factor A. Hepatic fibrosis is a chronic liver disease associated with an accumulation of extracellular proteins where excessive connective tissue builds up in the liver. The trigger could be accumulation of bile acids, glucose and free fatty acids and involvement of inflammatory component (Li and Apte 2015). Thus the indications of liver damage cannot be ruled out with an independent set of protein data in the same animal model.

### Integrated gene metabolite interactions

Expression profiling studies (Hammamieh et al. 2016) contributed significantly to the understanding of molecular mechanisms and when combined with metabolomics they have the potential to provide additional biological insight for understanding of disease pathogenesis. For further exploration of potential molecular connections of the identified metabolites with disease progression mechanisms, we combined our metabolomics dataset with our previously published (Hammamieh et al. 2016) dataset of 1,176 genes that were differentially expressed across time points. We understand that this analysis means fewer differentially changed metabolites as compared to genes and is likely to overlook some of the metabolite information. We are able to build gene/metabolite centric maps presented as interaction networks (Fig. 4b) to represent significant interactions among differentially expressed genes and altered metabolites. From these two data types, we identified a highly connected and integrated network of fatty acids and transcripts. Two key fatty acids, linoleate and (5Z–8Z–11Z–14Z) icosatetraenoic acid, showed regulatory interactions (integrated either directly or indirectly) with 73 upregulated and 80 downregulated transcripts which seemed to regulate every other metabolite in the network. As early as 6 h post infection, the metabolites icosatetraenoic acid and myo-inositol three phosphate along with the gene prostaglandin-endoperoxide synthase 2 (*ptgs2*) were observed as main nodes. The *ptgs2* genes encodes a key enzyme in prostaglandin biosynthesis, and is highly inducible following pro-inflammatory stimuli such as cytokines or endotoxins (Gagnaire et al. 2016). The metabolite linoleate showed up as a dominant node at 12 h post infection. Multiple *cyp* genes from the Cytochrome P450 superfamily and *pla* genes from phospholipase A2 family showed up as prominent nodes 18 h onwards post- infection. Linoleate and (5Z,8Z,11Z,14Z)-icosatetraenoic acid are highly connected to the Cytochrome P450 superfamily, phospholipase A groups and arachidonate lipoxygenase proteins. These proteins are important in metabolism of linoleic and icosateraenoic acids. Notably, these pathways were centered around lipid derived mediators related to arachidonic acid metabolism. Particularly, differentially increased synthesis of the pro-inflammatory fatty acid, linoleic acid (Burns et al. 2018), seems to have an important implication with regard to disease progression due to *Y. pestis* infection. Fatty acids are important sources of energy and the association of up and down regulated transcripts (which formed part of the network) is important information that could be responsible for accumulation of metabolites in response to *Y. pestis* infection (insults).

## Conclusions

Metabolomics proved to be an ideal technology to assess the effects of pneumonic *Y. pestis* infection in NHPs. An early inflammatory response was suggested by increases in arachidonic acid, omega-6 fatty acid precursors, and monohydroxy fatty acids (13- and/or 9-HODE). This study is novel because no other studies have identified metabolites that are involved in the course of infection of pneumonic plague. Also, in support of an early inflammatory response were indications of oxidative stress with decreases in α- and β-tocopherol and increases in biliverdin and bilirubin.

**Fig. 5:**
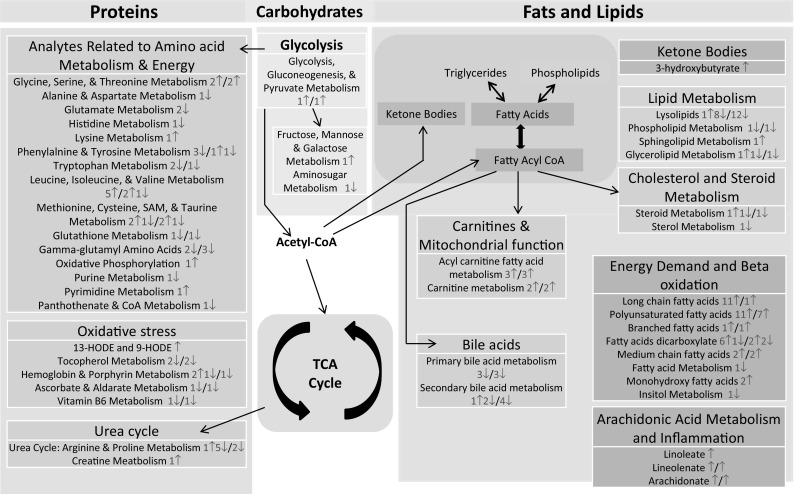
Summary overview: major metabolic pathways active in host: The color-coded boxes represent matching functional units. The red and blue numbers are the numbers of metabolites from early and late time points, respectively. The up and down arrows next to the numbers indicate the significantly increased and decreased levels of metabolites, respectively

The other major finding in this study was an increased energy demand on the host. A rapid mobilization of fatty acids and subsequent β-oxidation of these free fatty acids indicated a need for increased energy. The formation of ketone bodies and α-hydroxybutyrate revealed increased and potentially overwhelmed mitochondrial functions. The oxidation of omega fatty acids and branched chain amino acids also supports the hypothesis that the high energy demands of the host response to the infection are met through multiple mechanisms. These metabolic changes could lead to the general lack of energy that hosts display shortly after becoming infected with the pathogen but prior to expressing overt symptoms. Figure 5 shows a summary of the metabolically active pathways in the host, including the fats/lipids and amino acid pathways. Based on the data, we hypothesize there was dysfunction in the liver tissue that warrants further investigation. Potential next steps include the examination of multiple tissues of the infected animals to fully characterize the molecular course of infection.

## Electronic supplementary material

Below is the link to the electronic supplementary material.

Supplementary material 1 (PPTX 78 KB)

Supplementary material 2 (XLSX 594 KB)

Supplementary material 3 (XLSX 18 KB)

## Data Availability

The metabolomics metadata is reported in this paper and is available as supplement Table 1.
