# Supplementary material for: Metabolomic analyses reveal lipid abnormalities and hepatic dysfunction in non-human primate model for Yersinia pestis
Source: Metabolomics. 2018 Dec 29;15(1):2. doi: 10.1007/s11306-018-1457-2 (PMC6311182; doi:10.1007/s11306-018-1457-2)
Supplement: Supplementary file 1 — Supplementary material 1 (PPTX 78 KB) [file 11306_2018_1457_MOESM1_ESM.pptx]

## Slide 1
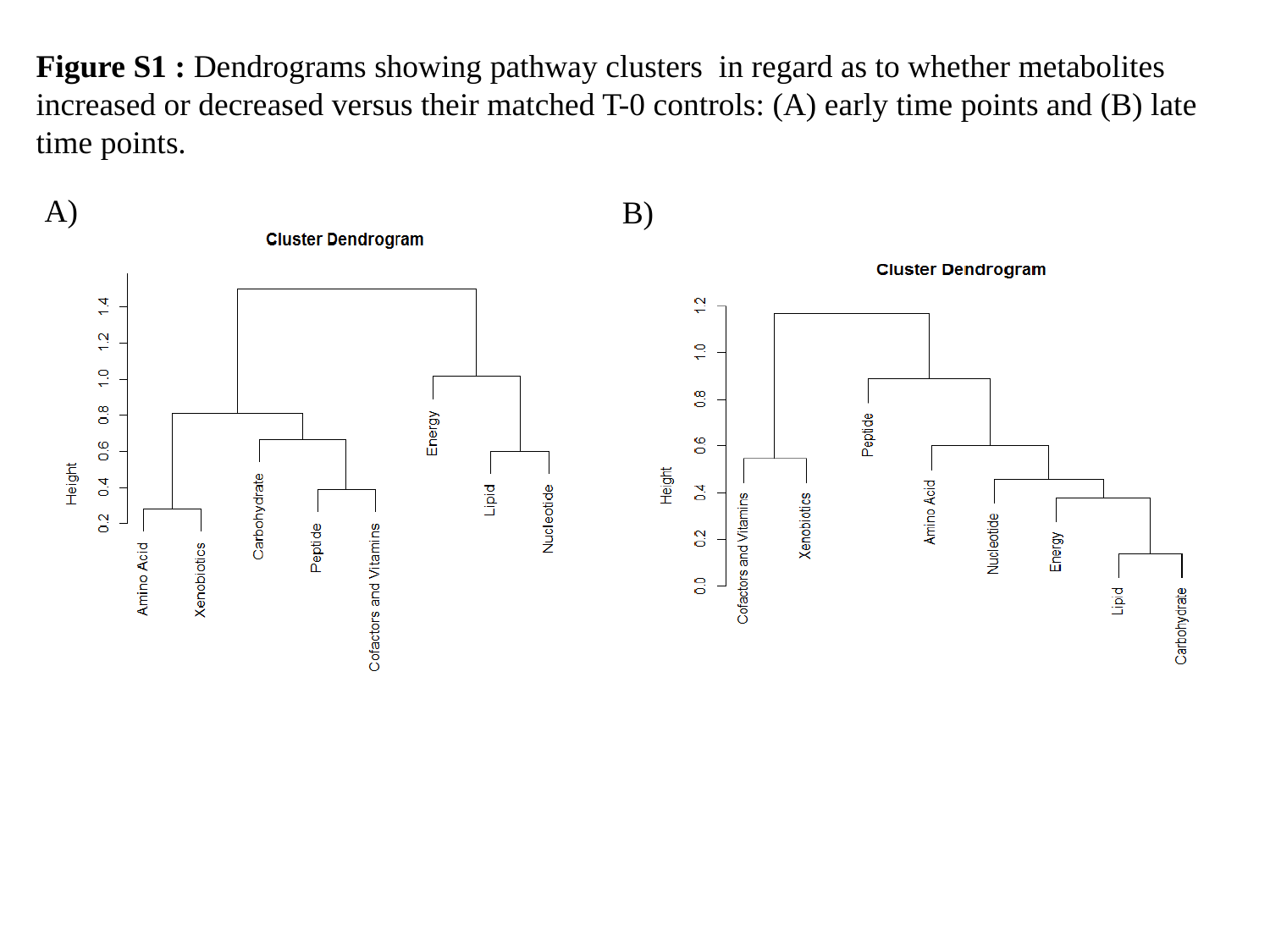

Figure S1 : Dendrograms showing pathway clusters in regard as to whether metabolites increased or decreased versus their matched T-0 controls: (A) early time points and (B) late time points.
A)
B)
